# Observations on the Asiatic Cholera, Etc.

**Published:** 1854-08

**Authors:** 


### BOOK NOTICES.

*Observations on the Asiatic Cholera as it appeared in Cincinnati in the years* 1849 *and* 1850. By THOS. CARROLL, M.D. Reprinted from the Western Lancet for June, 1854.

THIS is a monograph of 75 pages, giving a somewhat detailed account of the appearance, progress, and treatment of epidemic cholera in Cincinnati during the years 1849—50. It is very well written, and contains many facts in relation to the progress and results of the disease which are both interesting and valuable. The author is a firm believer in the contagiousness of the disease, and seems to adopt the popular idea that it originated in Asia and actually travelled (or its efficient cause) from thence across Europe and the Atlantic to America. He refuses to admit any valid or practical distinction between infection and contagion. We do not perceive that Dr. Carroll advances anything new in relation either to the pathology or treatment of Cholera. His views of the latter seem to us generally judicious, though he uses *brandy* much more than we think necessary or beneficial. There is probably no one remedy which has been so universally tried in the treatment of Cholera, as "pure brandy," so-called. We have studied its effects carefully, and are so well satisfied that it is entirely useless, if not positively injurious, in the great majority of cases, that it has been almost entirely discarded from our practice during the last three epidemics through which we have passed. As stimulants to support the circulation and temperature of the system, alcoholic drinks are much less efficient than tea and coffee, while their secondary effects on the blood and tissues are positively injurious.

The remedies on which Dr. Carroll chiefly relies are, in the first stage, alteratives and tonics; and in the second or active stage, calomel and opium, given in small and frequently repeated doses. He prefers giving one or two grains of calomel, with from the tenth to the twentieth of a grain of opium, every five or ten minutes, rather than larger doses at longer intervals. In this he follows pretty closely the plan of Dr. Ayres of England.

